# Hsp20 Functions as a Novel Cardiokine in Promoting Angiogenesis *via* Activation of VEGFR2

**DOI:** 10.1371/journal.pone.0032765

**Published:** 2012-03-12

**Authors:** Xiaowei Zhang, Xiaohong Wang, Hongyan Zhu, Evangelia G. Kranias, Yaoliang Tang, Tianqing Peng, Jiang Chang, Guo-Chang Fan

**Affiliations:** 1 Department of Pharmacology and Cell Biophysics, University of Cincinnati College of Medicine, Cincinnati, Ohio, United States of America; 2 Department of Internal Medicine, University of Cincinnati College of Medicine, Cincinnati, Ohio, United States of America; 3 Critical Illness Research, Lawson Health Research Institute, Ontario, Canada; 4 Institute of Biosciences and Technology, Texas A&M Health Science Center, Houston, Texas, United States of America; Northwestern University, United States of America

## Abstract

Heat shock proteins (Hsps) are well appreciated as intrinsic protectors of cardiomyocytes against numerous stresses. Recent studies have indicated that Hsp20 (HspB6), a small heat shock protein, was increased in blood from cardiomyopathic hamsters. However, the exact source of the increased circulating Hsp20 and its potential role remain obscure. In this study, we observed that the circulating Hsp20 was increased in a transgenic mouse model with cardiac-specific overexpression of Hsp20, compared with wild-type mice, suggesting its origin from cardiomyocytes. Consistently, culture media harvested from Hsp20-overexpressing cardiomyocytes by Ad.Hsp20 infection contained an increased amount of Hsp20, compared to control media. Furthermore, we identified that Hsp20 was secreted through exosomes, independent of the endoplasmic reticulum-Golgi pathway. To investigate whether extracellular Hsp20 promotes angiogenesis, we treated human umbilical vein endothelial cells (HUVECs) with recombinant human Hsp20 protein, and observed that Hsp20 dose-dependently promoted HUVEC proliferation, migration and tube formation. Moreover, a protein binding assay and immunostaining revealed an interaction between Hsp20 and VEGFR2. Accordingly, stimulatory effects of Hsp20 on HUVECs were blocked by a VEGFR2 neutralizing antibody and CBO-P11 (a VEGFR inhibitor). These *in vitro* data are consistent with the *in vivo* findings that capillary density was significantly enhanced in Hsp20-overexpressing hearts, compared to non-transgenic hearts. Collectively, our findings demonstrate that Hsp20 serves as a novel cardiokine in regulating myocardial angiogenesis through activation of the VEGFR signaling cascade.

## Introduction

It is well appreciated that heat shock proteins (Hsps) are activated in the mammalian heart in response to numerous physiological or pathological stresses and consequently, provide cardioprotection [1], [2]. Hsp20, also referred to as HspB6, belongs to a small heat shock protein family (15–30 kDa) which includes at least 10 members (HspB1–B10) [3], [4]. While Hsp20 can be detected in various tissues, it is most highly expressed in muscle cells [3]–[5]. Over the past years, our laboratory has shown that elevated intracellular Hsp20 protects hearts against various stress stimuli including myocardial ischemia/reperfusion (I/R) injury [6], isoproterenol-triggered cardiac remodeling [7], endotoxin-induced myocardial dysfunction [8], and doxorubicin cardiotoxicity [9]. These salutary effects of Hsp20 are largely attributed to the inhibition of cardiomyocyte death through multiple interactions with α-actin, α-actinin, Akt, Bax, NF-κB, 14-3-3γ, phosphodiesterase-4 (PDE4), and apoptosis signal-regulating kinase 1 (ASK1) [6]–[14]. Interestingly, we showed that Hsp20-engineered mesenchymal stem cells (MSCs) augmented the secretion of growth factors (VEGF, FGF-2, and IGF-1) and promoted myocardial angiogenesis [15]. However, the mechanistic role of Hsp20 in cardiac angiogenesis remains obscure.

Recently, a growing number of proteins are found to be secreted from the heart [16], [17]. These heart-derived proteins are now termed cardiokines [17]. Several experimental approaches have estimated the number of putative cardiokines to be between 30 and 60 [17]. Importantly, the cardiokines identified so far have been shown or predicted to play critical roles in maintaining normal cardiac development and potential repair of damaged/diseased myocardium [17]. Numerous studies indicate that some Hsps (i.e. Hsp90, Hsp70, Hsp60, and αB-crystallian) are detectable outside a variety of cell types including neuronal cells, monocytes, macrophages, endothelial cells and tumor cells of epithelial origin [18]–[22]. However, whether Hsps serve as cardiokines is not well defined. Notably, Hsp20 is detectable in blood and is believed to inhibit platelet aggregation [23]. A study by Kozama et al. [24] further demonstrated that plasma Hsp20 levels were increased in cardiomyopathic hamsters. Nonetheless, it remains unclear whether circulating Hsp20 is mainly derived from cardiomyocytes or from endothelial cells of the coronary vasculature.

In this study, we utilized a mouse model with cardiac-specific overexpression of Hsp20 to decipher whether circulating Hsp20 is increased. For the first time, we demonstrated that Hsp20 was secreted from adult rat cardiomyocytes via exosomes, independent of the classical ER-Golgi protein export pathway. Moreover, we identified what we believe to be a novel function for the extracellular Hsp20 in hearts as a mediator of angiogenesis through directly interaction with VEGFR2.

## Materials and Methods

### Mouse Model, Cell lines, Adenovirus Vectors

All animal protocols conformed to the *Guidelines for the Care and Use of Laboratory Animals* prepared by the National Academy of Sciences and published by the National Institutes of Health, and were approved by the University of Cincinnati Animal Care and Use Committee (Animal Welfare Assurance Number: A3295-01). A transgenic mouse model with cardiac-specific overexpression of Hsp20 and a recombinant adenovirus vector Ad.Hsp20 were described previously [6], [12]. HEK293 cells (human embryonic kidney cell line) purchased from the ATCC (Manassas, VA) were cultured in DMEM supplemented with 10% FBS and 100 µg/ml penicillin/streptomycin. The HUVECs (human umbilical vein endothelial cell) (PromoCell, Germany) were maintained in Endothelial Cell Growth Media with supplementMix and 100 µg/ml penicillin/streptomycin, which did not contain VEGF.

### *In vivo* Ischemia/Reperfusion

Ischemia/reperfusion was performed in male adult mice (FVB/N, 10–12 weeks old) by transiently ligating the left anterior descending coronary artery (LAD) for 30 min, followed by 24 hours of reperfusion [6]. Blood samples were collected for further experiments. Hearts were fixed in 4% paraformaldehyde and embedded in paraffin for morphological analysis.

### ELISA for the Detection of Extracellular Hsp20

Hsp20 in sera or supernatants was detected by an optimized sandwich ELISA. Briefly, high-binding 96-well ELISA plate (BD Biosciences) was coated overnight with rabbit polyclonal anti-Hsp20 (Affinity Bioreagents) in carbonate buffer. Samples or recombinant mouse Hsp20 protein (EIAab Science) were added and incubated for 2 h followed by three washes in PBST. Mouse monoclonal anti-Hsp20 (Fitzgerald) was used as a detection antibody and incubated for 2 h followed by five washes. Finally, TMB substrate reagent (BD Biosciences) was added, and absorbance was read by a microplate reader (Biotek) at 450 nm.

### Immunofluorescence Staining

Heart sections (3 µm) were incubated with specific two-mixtures of Abs: mouse anti-Hsp20 (Fitzgerald)/rabbit anti-VEGFR2 (Acris) for detection of colocalization; rabbit anti-Hsp20 (R&D)/mouse anti-α-Actin (Sigma-Aldrich) for detection of Hsp20 translocation; and rabbit anti-CD31 (Abbiotec)/mouse anti-α-Actin antibody for detection of vascular density. Specific binding of primary Abs was detected using corresponding Alex488- or Alex594-conjugated secondary Abs (Invitrogen). Immuno-staining was examined using an Olympus BX41 fluorescence microscope (Olympus America, Melville, NY), and evaluated using IPP 5.1 and MagnaFire 2.1 software.

### Preparation of Adult Rat Cardiomyocytes and Lactate Dehydrogenase Assay

Rat ventricular myocytes were isolated from adult male Sprague-Dawley rats (6–8 weeks old) and plated on laminin-coated glass coverslips or dishes, as described previously [7]–[9], [12], [25]. After 2 h, attached cardiomyocytes were infected with Ad.GFP (control) or Ad.Hsp20 in diluted media, at a multiplicity of infection (MOI) of 500, for 2 h before addition of suitable volume of culture media. For isolation of exosomes, culture media were collected after 48 h of adenoviral infection, when the infection efficiency reached more than 95%, determined by GFP under fluorescence microscopy. Cardiomyocyte injury was assessed by measuring Lactate dehydrogenase (LDH) release using an LDH detection kit, according to the manufacturer's instructions (Sigma-Aldrich).

### Isolation and Quantitation of Exosomes

Exosomes were collected on ice from 2 ml of adult rat cardiomyocytes media, using the Exosome precipitation kit (System Biosciences) according to the manufacturer's instructions. The amount of released exosomes was quantified by measuring the activity of acetylcholine esterase, an enzyme that is specifically involved in these vesicles [26]. Acetylcholine esterase activity was assayed as described previously [27]. All samples were plated in triplicate. The value represents the enzymatic activity after 20 min of incubation.

### HUVEC Proliferation, Migration and Tube Formation Assays

The assessment of cell proliferation was performed by MTS assay (CellTiter 96 AQueous One Solution Cell Proliferation Assay Kit, Promega), as the manufacturer's instructions. HUVECs were seeded into 96-well plates at an initial density of 5×10^3^ cells/well. After 2 h, recombinant human Hsp20 protein (US Biological) was added at various doses for 18–48 h. BSA was added as control. A curve of cell proliferation was constructed by measuring cell growth with a microplate reader at 490 nm.

The migration potency of HUVECs was determined by migration assays using the BD Falcon Cell Culture Insert System (BD Biosciences), according to the instructions of the manufacturer. We used a double-chamber method to determine the effect of recombinant human Hsp20 protein on HUVEC migration. HUVEC cells (5×10^4^ cells/well) were seeded into the upper transwell chambers with 8 µm pores, which were then placed into a 24-well plate in which the Hsp20 protein or medium only (control) were added. After 6 h of incubation, cells were stained with Hoechst 33342 (Invitrogen) for 30 min and the upper surface of transwell chambers was wiped with a cotton swab. Migrating cells were counted in five random microscopic fields (×200).

The formation of capillary-like structures was assessed in a 24-well plate using growth factor-reduced Matrigel (BD Biosciences). For this procedure, HUVECs (3×10^4^ cells/well) were plated on top of Matrigel (500 µl/well) 2 hours before recombinant human Hsp20 protein was added. After 16 h, cells were fixed in 70% ethanol and the total tube length was quantified using software Image-Pro plus 5.1.

### VEGFR2/Hsp20 Competitive Binding Assay

The purified VEGFR2 protein (ProSpec) and control protein (BSA) were diluted to a final concentration of 20 µg/ml with carbonate coating buffer and a high-binding 96-well ELISA plate was coated overnight at 4°C with 100 µl of the protein solution. The solution was removed followed by three washes with PBST. Then, the plate was incubated with 200 µl of blocking buffer (1% BSA) at room temperature for 1 h. Recombinant human Hsp20 protein was diluted at various concentrations (1.2, 2.4 and 4.8 µg/ml) with PBS. Plate was washed three times with PBST and 100 µl Hsp20 preparations were added and incubated at room temperature for 2 h. Plate was then washed three times before anti-Hsp20 antibody (1∶5000 dilution in PBS, Fitzgerald) was added. After 2 h incubation at room temperature followed by three washes, anti-mouse HRP conjugated secondary antibody (diluted 1∶5000 in PBS) was added and the plate was incubated for 1 h at room temperature. The plate was washed five times with PBST and 100 µl of TMB substrate reagent was added followed by a 25-min incubation at room temperature. The plate was read by a microplate reader (Biotek) at 450 nm as soon as 50 µl stop solution was added.

### Western Blot Analysis

Protein samples were extracted from hearts or cultured HUVECs, with procedures as described in detail elsewhere. Equal amounts of protein were subject to SDS-PAGE. Binding of the primary antibody was detected by peroxidase-conjugated secondary antibodies and enhanced chemiluminescence (Amersham), and bands were quantified with densitometry. The source of antibodies and dilutions used were as follows: mouse anti-Hsp20 antibody (1∶500 dilution, Fitzgerald), mouse anti-p-Akt (1∶500 dilution), mouse anti-Akt (1∶500 dilution), rabbit anti-p-ERK (1∶500 dilution), and rabbit anti-ERK (1∶500 dilution) antibodies (Cell Signaling). α-Actin (Sigma-Aldrich) or β-actin (Cell Signaling) was used as an internal control.

### Statistical Analysis

Data are presented as mean ± SD. Comparisons were made by Student's t test as appropriate. A value of P<0.05 was considered statistically significant.

## Results

### Hsp20 can be actively released from the myocardium *in vivo*

**Figure 1:**
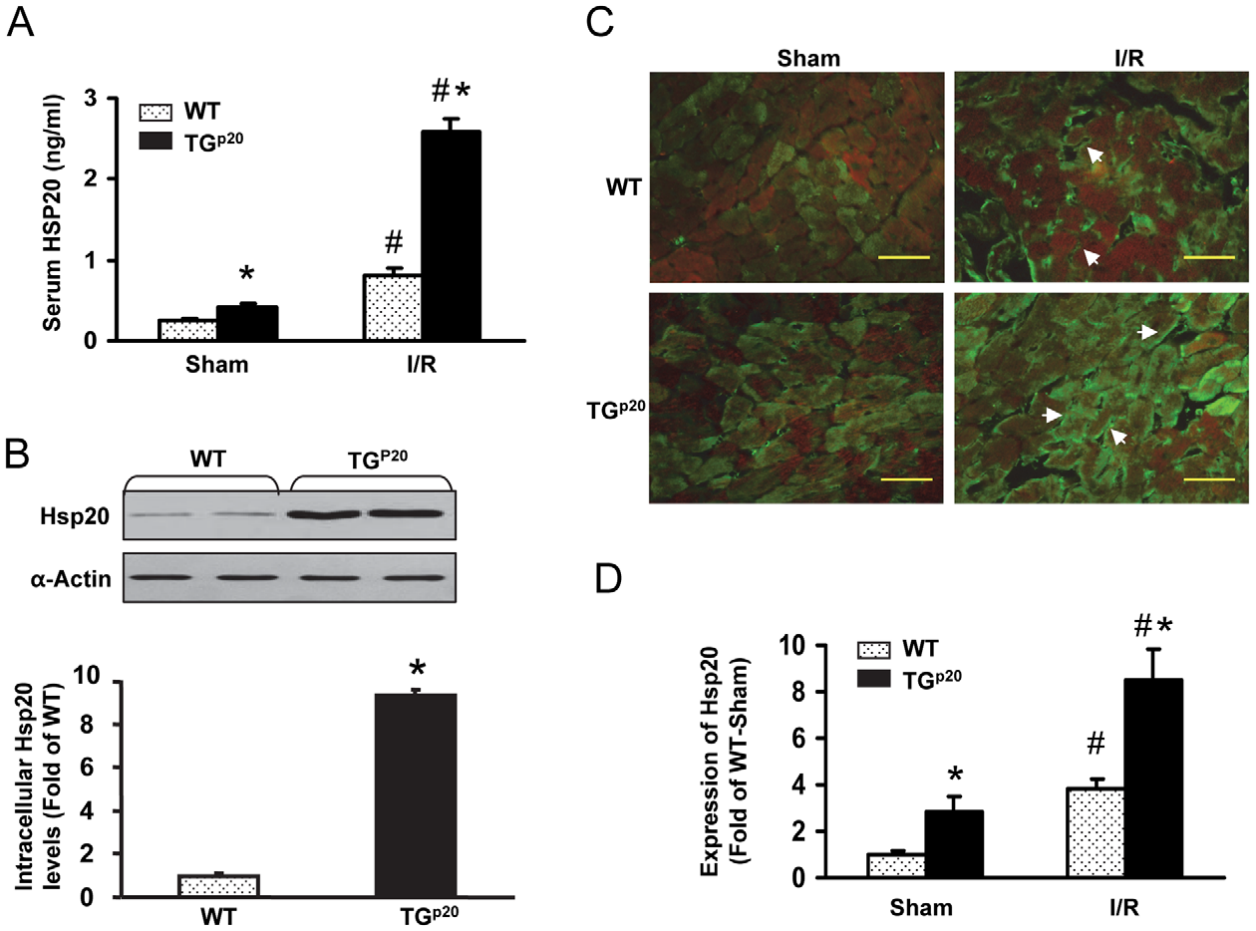
Hsp20 is secreted from cardiomyocytes *in vivo*. (A) The serum Hsp20 level was increased in response to *in vivo* 30 min-LAD occlusion followed by 24 h-reperfusion. Cardiac-specific overexpression of Hsp20 increased the Hsp20 concentration in the serum under basal and myocardial ischemia/reperfusion conditions. (n = 6; *, p<0.05 *vs.* WTs; #, p<0.05 *vs.* Sham groups). (B) The levels of Hsp20 in hearts from Hsp20-transgenic mice were determined by Western blot. α-Actin was used as an internal control (n = 4). (C) Myocardial ischemia/reperfusion stimulated the translocation of Hsp20 to the cardiomyocyte membrane, which was detected by fluorescence microscopy. Images are representative sections from four mice per group (green, Hsp20; red, α-Actin; Scale bar, 100 µm). (D) Quantitative data for expression of Hsp20 was evaluated using IPP 5.1 (n = 4; *, p<0.05 *vs.* WTs; #, p<0.05 *vs.* Sham groups).

It has been shown that plasma levels of Hsp20 in cadrdiomyopathic hamsters were markedly elevated [24]. To investigate whether circulating Hsp20 is increased in animals subjected to myocardial ischemia/reperfusion (I/R), 12-weeks old mice (FVB/N) underwent *in vivo* 30-min myocardial ischemia, via coronary artery occlusion, followed by 24-h reperfusion. Subsequently, sera were collected and levels of Hsp20 were measured using an optimized Sandwich ELISA. As expected, levels of circulating Hsp20 were increased by 3.4-fold in mice upon myocardial I/R insults, compared to the sham operation group (Figure 1A). Given that myocardial ischemia/reperfusion causes cell damage, the increased circulating Hsp20 may be derived from the injured myocardium. Therefore, it is still uncertain whether Hsp20 is passively or actively secreted from cardiomyocytes or endothelial cells.

**Figure 2:**
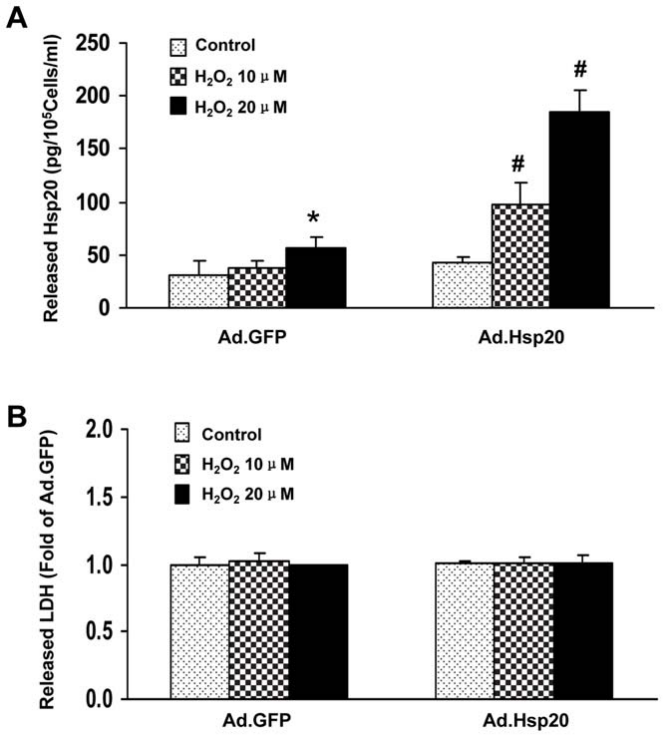
Overexpression of Hsp20 in adult cardiomyocytes enhances its secretion. (A) Mild stress dose-dependently increased the amount of Hsp20 released from both Ad.GFP and Ad.Hsp20 infected myocytes, and (B) there was no difference in the release of lactate dehydrogenase (LDH), a marker of necrosis. Similar results were observed in two additional, independent experiments (*, p<0.05 vs. Ad.GFP-Control; #, p<0.05 *vs.* Ad.GFP).

Hence, to clarify the origin of circulating Hsp20, we employed a transgenic (TG) mouse model with cardiac-specific overexpression of Hsp20 (10-fold, Figure 1B). Our results indicate that serum levels of Hsp20 was increased by 57% in the Hsp20 TG mice, compared to wild-type (WT) mice (Figure 1A). These data suggest that intracellular Hsp20 can be actively released outside cardiomyocytes into the bloodstream. Consistently, we observed that serum Hsp20 was dramatically elevated by 2.2-fold in Hsp20-TG mice upon myocardial I/R stress, compared with WT controls (Figure 1A). Furthermore, we determined the distribution of Hsp20 in hearts upon I/R. Immunostaining results demonstrated that myocardial I/R induced accumulation of Hsp20 on the membrane of cardiomyocytes with more significant in TGs, compared to WT samples (Figure 2C and D). These results indicate that enhanced expression of Hsp20 in the heart (genetically or pathologically) augments extracellular Hsp20.

### Hsp20 is secreted from cultured adult cardiomyocytes *in vitro*

To confirm our *in vivo* findings that elevated intracellular Hsp20 can be secreted outside cells, we performed further studies using *in vitro* cultured adult rat ventricular myocytes in a well controlled experimental setting. Adult rat cardiomyocytes were infected with Ad.Hsp20 and 48 h later, 10 or 20 µM of H_2_O_2_ was added to these myocytes for 2 h. Subsequently, culture supernatant was harvested for measuring the Hsp20 content. We observed that there was a small amount of Hsp20 released from unstressed cardiac myocytes, and the amount was too low to tell the difference between Ad.GFP-cells (31 pg/10^5^ cells/ml) and Ad.Hsp20-cells (40 pg/10^5^ cells/ml) (Figure 2A). However, upon addition of a mild stress with a low dose of H_2_O_2_ (20 µM) for 2 h, the content of Hsp20 in the culture supernatant was significantly increased, with a more pronounced increase seen in Hsp20-overexpressing myocytes compared to basal conditions (Figure 2A). Extracellular Hsp20 was reached to 60 pg/10^5^ cells/ml in Ad.GFP-myocytes, and dramatically increased to 191 pg/10^5^ cells/ml in Ad.Hsp20-myocytes upon H_2_O_2_ stimulation (Figure 2A). Importantly, dosages of H_2_O_2_ we used did not cause any cytotoxic effects, because the concentration of lactate dehydrogenase (LDH), a marker of the cell injury, in culture media was no different among groups (Figure 2B). Together, these data confirm the *in vivo* findings showed that Hsp20 was actively secreted from cardiomyocytes.

### Hsp20 is released via exosomes, independent of the classic protein export pathway

**Figure 3:**
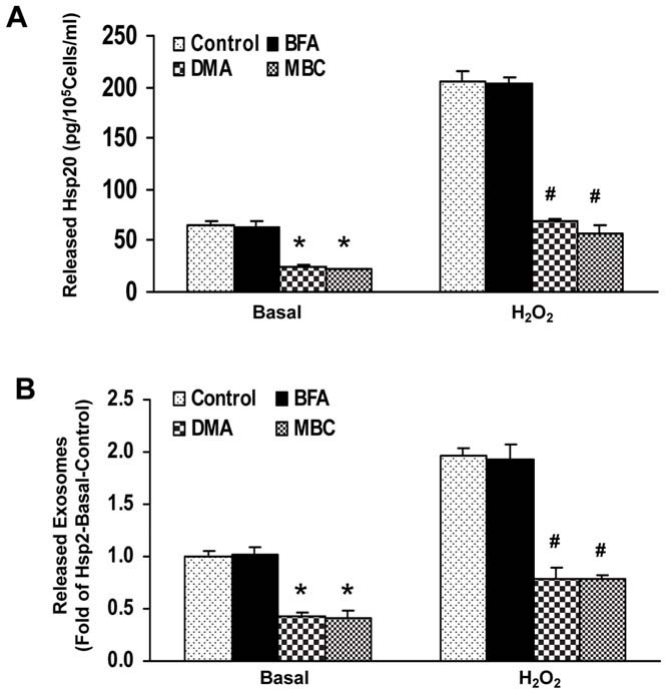
Hsp20 is secreted from adult rat cardiomyocytes *via* exosomes, independent of the ER-Golgi protein export pathway. (A) Brefeldin A (BFA), which inhibits the classical protein transport pathway, did not block Hsp20 release into the media under either basal or hypoxia conditions (20 µM H_2_O_2_). However, the release of Hsp20 from cardiomyocytes was reduced by both dimethyl amiloride (DMA), an exosome inhibitor, and Methyl-β-cyclodextrin (MBC), an inhibitor of lipid raft formation via depletion of membrane cholesterol. (B) The activity of acetylcholine esterase was used to quantify the amount of exosomes present in the media after various treatments. Similar results were observed in three additional, independent experiments (*, p<0.05 vs. Basal-Control; #, p<0.05 *vs.* H_2_O_2_-Control).

While Hsp20 is detectable outside cells, it remains elusive how to regulate the release of intracellular Hsp20. Currently, the endoplasmic reticulum (ER)/Golgi pathway is the most widely recognized mechanism for protein release from the cell [28]. However, accumulating evidence has established that several heat shock proteins (i.e. Hsp60, Hsp70 and αB crystalline) are secreted by a non-classical pathway that involves exosomes [26], [29], [30]. Therefore, we dissected both the classical ER-Golgi protein export pathway and alternative secretory pathway in Hsp20-overexpressing myocytes. Pre-treatment with 10 µg/ml Brefeldin A (BFA), an inhibitor of the classical protein transport pathway [26], [27], did not attenuate the Hsp20 secretion from cardiomyocytes under basal conditions (Figure 3A). Similarly, low dose H_2_O_2_ increased the release of Hsp20 into the media, which was not blocked by BFA treatment (Figure 3A). In contrast, the release of Hsp20 from cardiomyocytes was reduced by both dimethyl amiloride (DMA, an exosome inhibitor) and Methyl-β-cyclodextrin (MBC, an inhibitor of lipid raft formation via depletion of membrane cholesterol) under both basal and hypoxia conditions (Figure 3A). These results indicate that the release of Hsp20 is dependent on the formation of exosome/lipid raft.

To further validate the involvement of exosomes in the release of Hsp20, we isolated exosomes using the Exosome precipitation kit and measured acetylcholine esterase activity, an exosome marker. As shown in Figure 3B, both DMA and MBC inhibited the release of exosomes from cardiac myocytes under basal and hypoxic conditions, whereas BFA had no effect. These results indicated that the effect of these three inhibitors on exosome release parallels their effects on the release of Hsp20, suggesting that Hsp20 secretion is via exosomes.

### Extracellular Hsp20 promotes HUVEC proliferation, migration and tube formation

**Figure 4:**
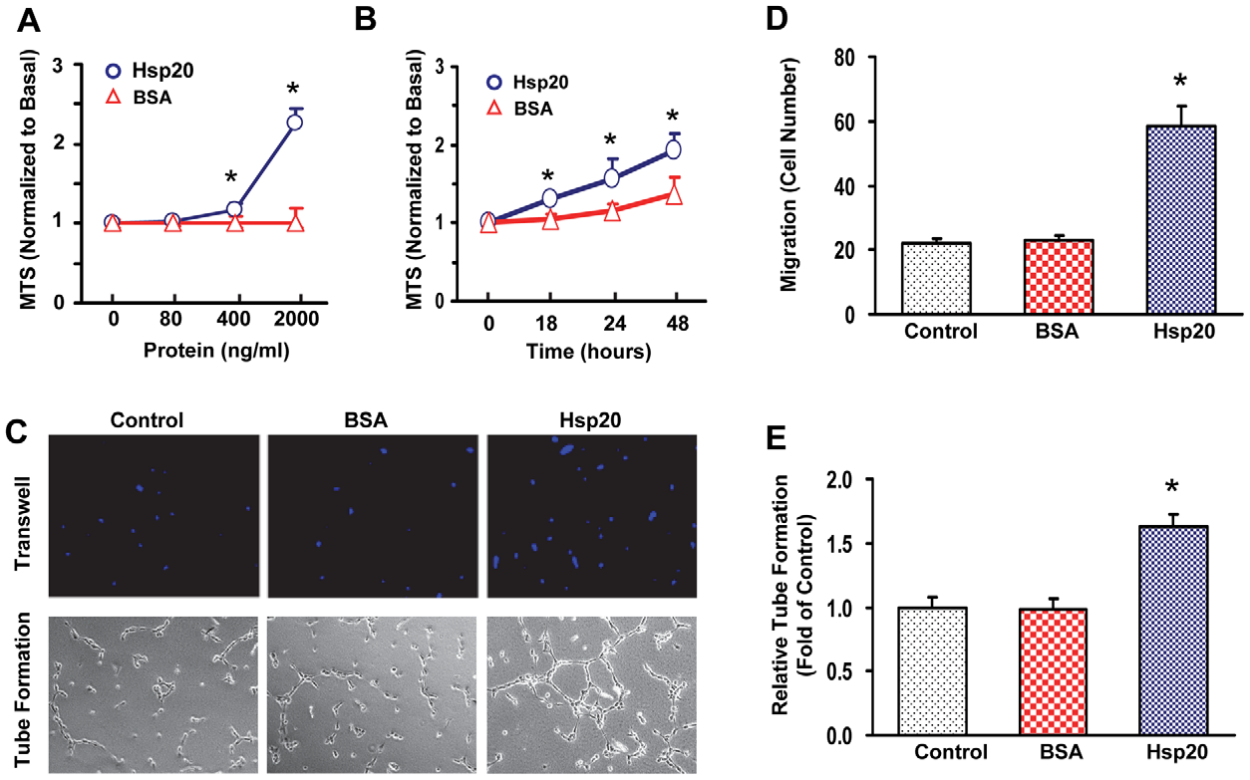
Hsp20 stimulates HUVEC proliferation, migration and capillary-like tube formation. (A) Recombinant human Hsp20 protein was added to HUVECs at various doses (80–2000 ng/ml) for 24 h. BSA was used as a control. Cell proliferation was determined by MTS. (B) Time-course effects of the Hsp20 protein(1000 ng/ml) on the HUVEC proliferation. (C) Representative photographs indicated the effects of recombinant human Hsp20 protein on the trans-well and tube formation of HUVECs. (D) Migration was quantified by counting cells that were moved through the membrane (Trans-well assay). (E) Tube formation was evaluated by the measurement of relative tube length. Similar results were observed in three additional, independent experiments (*, p<0.05 *vs.* Control).

Recently, our laboratory has showed that transplantation of Hsp20-engineered mesenchymal stem cells enhances the paracrine effects on infarcted hearts, as accompanied with increase in the vascular density [15]. However, it is unknown whether extracellular Hsp20 functions in cardiac paracrine signaling. Therefore, we employed human umbilical vein endothelial cells (HUVECs) to investigate whether extracellular Hsp20 promotes angiogenesis. A recombinant human Hsp20 protein was added to HUVECs at various doses (80, 400 and 2000 ng/ml) for 24 h, and BSA was used as a control. We observed that Hsp20 dose-dependently promoted the HUVEC proliferation, as measured by the MTS incorporation (Figure 4A). Furthermore, HUVECs were treated with the Hsp20 protein at a dose of 1000 ng/ml for 18, 24 or 48 h, the MTS proliferation-assay showed that Hsp20 could also time-dependently promoted the HUVEC proliferation (Figure 4B). Using a transwell assay, we found that recombinant human Hsp20 protein increased HUVECs migration by 2.6-fold compared with the controls. Importantly, no significant difference was observed between the BSA treatment and non-treated groups (Figures 4C upper-panel and 4D). Accordingly, the tube-like structures were dramatically enhanced by 63% with the addition of the Hsp20 protein, whereas the treatment with BSA yielded fewer tube-like structures (Figures 4C down-panel and 4E). Collectively, these data indicate that the extracellular Hsp20 can promote angiogenesis as evidenced by enhancing proliferation, migration and tube formation of endothelial cells.

### Extracellular Hsp20 promotes angiogenesis by binding with VEGFR2

**Figure 5:**
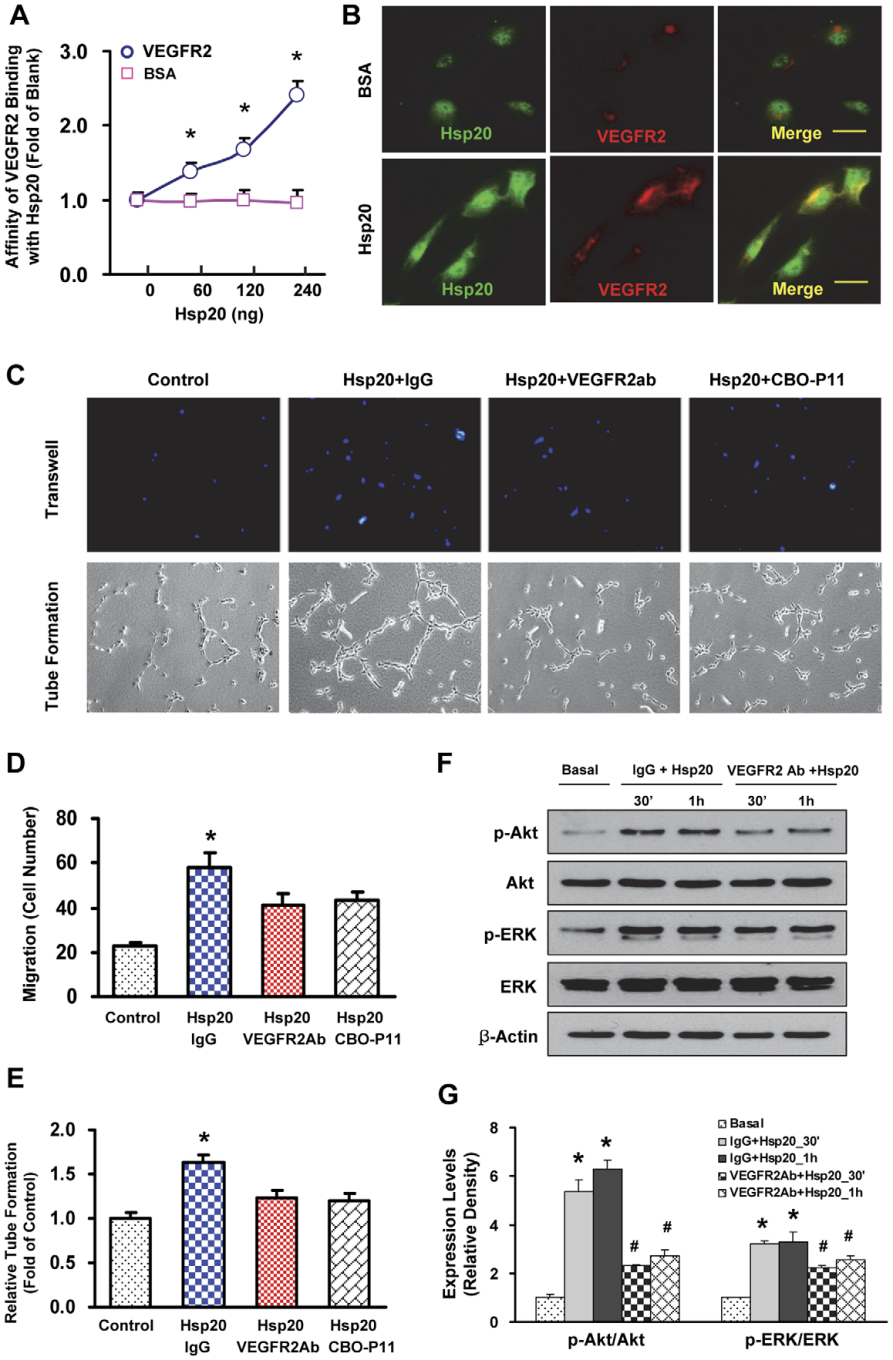
Extracellular Hsp20 interacts with VEGFR2 and activates its downstream signaling pathways. (A) VEGFR2 coated on a plate dose-dependently captured the Hsp20 protein, whereas BSA coated did not arrest the Hsp20 protein. (B) Recombinant human Hsp20 protein significantly induced the expression of VRGFR2 in HUVECs, and co-localized with VEGFR2 in the cell surface. Images are representative sections from 20 fields per group (green, Hsp20; red, VEGFR2). Scale bar, 25 µm. (C–E) Blockade of the VEGFR2 signaling by a VEGFR2 neutralizing antibody and CBO-P11 (a VEGFR inhibitor) suppressed the HUVEC migration (C and D) and tube formation (C and E). Similar results were observed in three additional, independent experiments (*, p<0.05 vs. Control). (F and G) Immunoblots determined the levels of Akt, p-Akt, ERK and p-ERK in Hsp20-treated HUVECs. IgG or VEGFR2 antibody was pre-treated 30 min prior to the addition of Hsp20. β-actin was used as an internal control (n = 4; *, p<0.05 *vs.* Basal; #, p<0.05 *vs.* IgG+Hsp20).

It is well appreciated that mitogenesis and migration of endothelial cells is mainly mediated by VEGFR2 [31]. Additionally, the Hsp20 protein has been predicated to contain several interactive domains with VEGF, FGF-2 and insulin [15]. Based on these data, we hypothesized that Hsp20 may interact with the receptors for these growth factors. To test this hypothesis, we first developed a competitive protein binding assay to examine whether Hsp20 binds with VEGFR2. Our results showed that VEGFR2-coated wells, but not BSA-coated wells, dose-dependently captured the Hsp20 protein, which were detected by an anti-Hsp20 antibody (Figure 5A). Moreover, immuno-fluorescence staining revealed that the addition of purified Hsp20 protein not only induced the expression of VEGFR2, but also co-localized with VEGFR2 in HUVECs (Figure 5B). Together, these data indicate that Hsp20 may directly interact with VEGFR2.

To further determine whether Hsp20-mediated actions on endothelial cells are dependent on VEGFR2, we pre-incubated HUVECs with a VEGFR2 neutralizing antibody or CBO-P11, a specific VEGFR inhibitor [32], for 30 min, followed by addition of the Hsp20 protein. We observed that both the VEGFR2 antibody and COP-P11 markedly reduced the stimulatory effect of Hsp20 on cell migration by 30% and 26%, respectively (Figure 5C- up panel and Figure 5D), compared with the IgG control. Consistently, the activity of Hsp20 on promoting endothelial cell tube formation was attenuated by pre-treatment with the neutralizing antibody or CBO-P11 (Figure 5C-downpanel and Figure 5E). Taken together, these results demonstrate that the effect of Hsp20 on the HUVEC migration and tube formation is largely dependent on VEGFR2.

Considering that the activation of Akt and ERK pathways plays a critical role in VRGFR2-mediated endothelial cell phenotype [31], we next examined whether extracellular Hsp20 activates the VEGFR2-mediated downstream signaling. Quantitative results of western blots showed that levels of phosphorylated Akt were increased by ∼6-fold in HUVECs at 30 min to 1 hour after treatment with the Hsp20 protein, which were significantly inhibited by the VEGFR2 neutralizing antibody (Figure 5F and 5G). Likewise, levels of phosphorylated ERK were also elevated at 30 min to 1 hour, but decreased by ∼30% with pre-treatment of the VEGFR2 antibody before the addition of Hsp20 (Figure 5F and 5G). These results suggest that Hsp20-mediated angiogenesis is largely associated with the Akt and ERK signaling pathways via activation of VEGFR2.

### Cardiac-specific overexpression of Hsp20 increases capillary density

**Figure 6:**
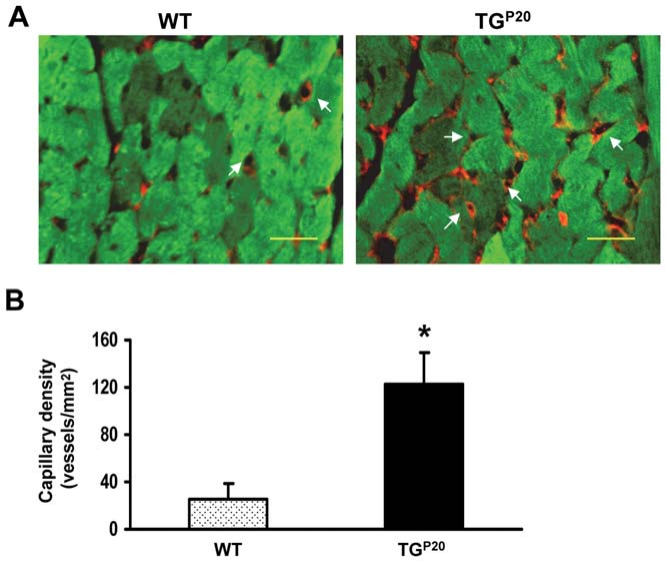
Cardiac-specific overexpression of Hsp20 promotes myocardial angiogenesis. (A) Blood vessels were stained for CD31 (capillary density) in heart sections of WT and Hsp20 TG mice, and (B) their quantitative analysis. For quantification of positively stained vessels, five sections of each heart (n = 4 hearts per group) were analyzed by an investigator who was blinded with respect to samples. Blood vessels were detected at low magnification (×200). Images are representative sections from four mice per group (green, α-Actin; red, CD31). Scale bar, 50 µm.

**Figure 7:**
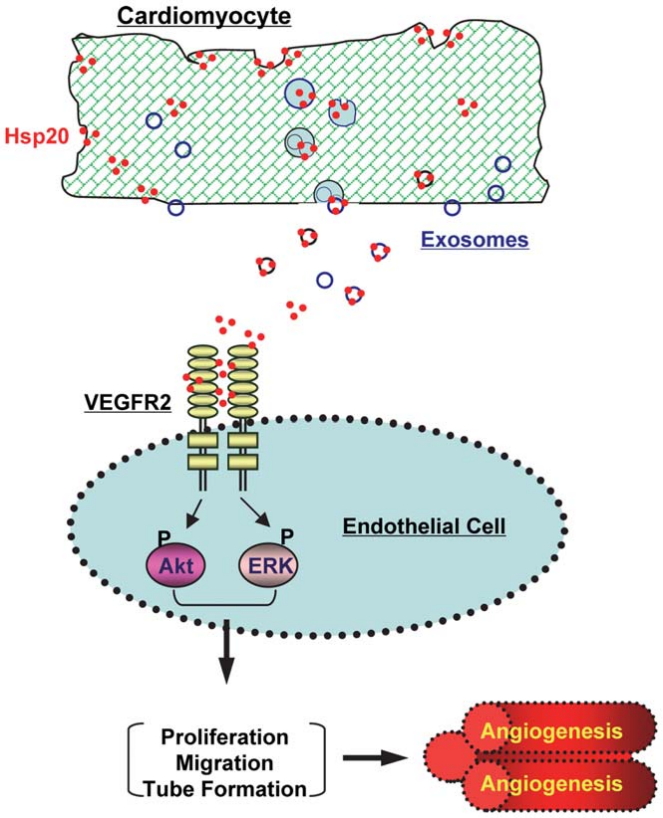
Proposed mechanism of the Hsp20 release and its regulation of myocardial angiogenesis. Intracellular Hsp20 is released outside cardiomyocytes via exosomes, and then interacts with VEGFR2. Consequently, its downstream signaling pathways (i.e. Akt and ERK) are activated, which promote myocardial angiogenesis.

Knowing that circulating Hsp20 was increased in Hsp20-TG mice, and extracellular Hsp20 promoted HUVEC proliferation, migration and tube formation, it is reasonable to speculate that the enhanced expression of intracellular Hsp20 could lead to an augmentation of myocardial angiogenesis. Therefore, we measured cardiac capillary density by immuno-fluorescence staining with an antibody to CD31, a specific marker of endothelial cells [15]. We observed that Hsp20 transgenic hearts revealed a significant increase in capillary density (122±28/mm^2^), compared to wild-type hearts (25±12/mm^2^) (Figure 6). These results clearly indicate that cardiac-specific overexpression of Hsp20 can promote myocardial angiogenesis through its secretion which functions *via* paracrine signaling (Figure 7).

## Discussion

Hsp20 is well recognized to act intracellularly as a molecular chaperone and confers protection against various hazardous conditions [3]–[6]. In the present study, we identified that Hsp20 was released from adult cardiomycoytes through exosome-dependent secretory machinery (Figure 7). Furthermore, we uncovered that extracellular Hsp20 physically bound to the VEGF receptor and thereby activated the downstream singling pathway (i.e. Akt, EKR). As a result, myocardial angiogenesis was enhanced in Hsp20-overexpressing hearts (Figure 7). To our knowledge, this is the first report to demonstrate that extarcellular Hsp20 functions as a VEGF-receptor agonist.

While intracellular Hsp20 was overexpressed by 10-fold in our cardiac-specific transgenic mice, the circulating Hsp20 was increased only 57%, compared to non-transgenic mice (Figure 1). This result indicates that most of Hsp20 is retained inside cells under the normal physiological state. However, upon hypoxic/ischemic stress, much more intracellular Hsp20 was translocated to the cellular membrane or packaged within exosomes (Figure 1) and subsequently secreted outside cardiomyocytes. Currently, although it is not clear how Hsp20 participates in the formation of exosomes, the membranous distribution of Hsp20 may largely contribute to the component of exosomes. Likewise, a recent study by Gupta et al. [26] also demonstrated that unstressed cardiomyocytes released Hsp60 but the addition of 2-hour hypoxia, increased the amount of extracellular Hsp60 without evidence of necrosis. These data imply that extracellular Hsps may be physiological alarm signals for myocardial infarction. Interestingly, while intracellular Hsp60 has an anti-apoptotic role in cardiomyocytes [33], extracellular Hsp60 can precipitate cardiac apoptosis through activation of Toll-like receptor (TLR)-4 [34]. By contrast, our previous studies [6]–[9], [12], [15] and current data demonstrate that Hsp20, regardless of location and despite different underlying mechanisms, consistently elicits a salutary effect in the heart.

Actually, overexpression of Hsp20 in the heart not only resists stress-triggered cardiomyocyte death via the intrinsic anti-apoptotic pathways, but also causes increased secretion of Hsp20 outside cardiomyocytes, which may function in autocrine or paracine signaling, enhancing the survival of cardiomyocytes and non-cardiomyocytes. Furthermore, the circulating Hsp20 has been demonstrated to bind to platelets and inhibit their aggregation [23], [24], which may be beneficial for the treatment of ischemic heart disease. In the present study, we identified a heretofore unrecognized role of the extracellular Hsp20 in cardiac angiogenesis. Given that coronary angiogenesis is instrumental in functional compensation and restoration of the heart [31], [32], increased capillary density in cardiac-specific Hsp20-overexpressing mice may provide a new mechanism underlying Hsp20-mediated protective effects against potential cardiac injury. Taken together, these data suggest that Hsp20 may serve as a novel cardiokine and benefit hearts at multiple levels.

Our study did not precisely address several clinical issues including: whether the Hsp20 protein is stable in the extracellular environment; whether the Hsp20 protein can be employed to treat myocardial infarction by exogenous systemic administration; and what advantage the Hsp20 protein might have over VEGF in promoting myocardial angiogenesis. Such analysis may fall outside the scope and intent of this report. However, the data presented here raise a novel hypothesis that Hsp20-containing exosomes may have a promising therapeutic application in the treatment of ischemic heart disease. In addition to the instrumental role of Hsp20 in the heart [3]–[15], a number of recent studies have implicated exosmes as intercellular signalsomes and pharmacological effectors [35]. First, exosomes are small, plasma-membrane-derived particles (usually 30–100 nm) that can be easily taken up by the surrounding cells [36]. Second, exosomes secreted from human mesenchymal stem cells have been shown to reduce myocardial ischemia/reperfusion injury in animal models [37], and cardiomyocyte progenitor cell-derived exosomes stimulated migration of endothelial cells [38]. Moreover, in mast cells, exosomes were demonstrated to communicate a protective signal to recipient cells during oxidative stress resulting in reduced cell death [39]. Therefore, it would be expected that Hsp20-containing exosomes may have combined effects to a great extent in both extrinsic and intrinsic protective pathways for the heart. Future studies are needed to clarify this hypothesis.

In conclusion, this study demonstrates that Hsp20, similar to other cardiokines, is actively released from cardiomyocytes under both steady state and stressed conditions *via* exosomes. Our findings highlight a novel role for Hsp20 in facilitating myocardial angiogenesis through interaction with VEGFR2, suggesting a possible novel therapeutic approach to inducing the angiogenic pathway by Hsp20 gene therapy or administration of Hsp20-containing exosomes.
